# Progress in Prediction and Interpretation of Clinically Relevant Metabolic Drug-Drug Interactions: a Minireview Illustrating Recent Developments and Current Opportunities

**DOI:** 10.1007/s40495-017-0082-5

**Published:** 2017-02-01

**Authors:** Stephen Fowler, Peter N. Morcos, Yumi Cleary, Meret Martin-Facklam, Neil Parrott, Michael Gertz, Li Yu

**Affiliations:** 1 Pharmaceutical Research and Early Development, Roche Innovation Centre Basel, F. Hoffmann-La Roche Ltd., Grenzacherstrasse 124, CH-4070 Basel, Switzerland; 2 Pharmaceutical Reseach and Early Development, Roche Innovation Center New York, F. Hoffmann-La Roche Ltd., 430 East 29th Street, New York City, NY USA

**Keywords:** Drug-drug interaction, Prediction, Physiologically based pharmacokinetic model, Metabolism, Regulatory submission, Cytochrome P450

## Abstract

**Purpose of Review:**

This review gives a perspective on the current “state of the art” in metabolic drug-drug interaction (DDI) prediction. We highlight areas of successful prediction and illustrate progress in areas where limits in scientific knowledge or technologies prevent us from having full confidence.

**Recent Findings:**

Several examples of success are highlighted. Work done for bitopertin shows how in vitro and clinical data can be integrated to give a model-based understanding of pharmacokinetics and drug interactions. The use of interpolative predictions to derive explicit dosage recommendations for untested DDIs is discussed using the example of ibrutinib, and the use of DDI predictions in lieu of clinical studies in new drug application packages is exemplified with eliglustat and alectinib. Alectinib is also an interesting case where dose adjustment is unnecessary as the activity of a major metabolite compensates sufficiently for changes in parent drug exposure.

Examples where “unusual” cytochrome P450 (CYP) and non-CYP enzymes are responsible for metabolic clearance have shown the importance of continuing to develop our repertoire of in vitro regents and techniques. The time-dependent inhibition assay using human hepatocytes suspended in full plasma allowed improved DDI predictions, illustrating the importance of continued in vitro assay development and refinement.

**Summary:**

During the past 10 years, a highly mechanistic understanding has been developed in the area of CYP-mediated metabolic DDIs enabling the prediction of clinical outcome based on preclinical studies. The combination of good quality in vitro data and physiologically based pharmacokinetic modeling may now be used to evaluate DDI risk prospectively and are increasingly accepted in lieu of dedicated clinical studies.

**Electronic supplementary material:**

The online version of this article (doi:10.1007/s40495-017-0082-5) contains supplementary material, which is available to authorized users.

## Introduction

Quantification of a drug-drug interaction (DDI) effect in a man is the basis for explicit dose recommendation in drug labels to minimize the risk of adverse events or reduced efficacy, thereby supporting appropriate use of the drug. It is therefore essential that such quantitative DDI assessments are made with confidence. There has been a steady development of in vitro assays and the reagents available for the study of drug metabolism and metabolic enzyme inhibition. This, combined with advances in our capability to extrapolate in vitro data to in vivo, has brought us past a “tipping point” such that applying a model-based synthesis of the available data has become normal in drug-drug interaction assessments [1–5••]. Simple static models, built upon DDI studies reaching back to the 1970s [6•], still find utility in early drug discovery where there are very limited data available for the drug candidate. However, the greatest DDI effects are observed where the metabolism of an orally administered drug is substantially inhibited in the first pass metabolism, potentially in both the intestine and liver. The combination of increased drug reaching the systemic circulation as well as reduced systemic clearance will result in a significantly higher exposure (area under the plasma concentration-time curve [AUC]) than when inhibition of systemic clearance alone is considered. An example of this can be seen when comparing the DDI effect of ketoconazole on alprazolam and midazolam which are low and high clearance cytochrome P450 (CYP) 3A substrates, respectively. In the recent study of Boulenc et al., peak concentration (C_max_) for alprazolam and midazolam were increased by 1.18- and 4.21-fold, whereas AUC was increased by 2.63- and 16.95-fold, respectively, when co-administered with multiple once-daily doses of 400 mg ketoconazole [7]. Mechanistic static models have significantly extended mathematical model usage, by incorporating additional considerations such as intestinal metabolism, enzyme induction, and enzyme inactivation [8, 9]. Nevertheless, these mathematical models cannot capture the full dynamic nature of drug metabolism in vivo since only a fixed concentration of inhibitor is considered. For example, DDI effects on simultaneous co-administration versus staggered dosing situations may be different, especially when the interacting drugs have short half-lives and high first pass metabolism. Details of the different approaches to DDI prediction were recently described in a Pharmaceutical Industry Innovation and Quality working group publication from Bohnert et al. and will not be discussed further in this review [10•].

A more powerful approach to DDI prediction can be taken using physiologically based pharmacokinetic (PBPK) modeling, especially when human pharmacokinetic data are available. Validated PBPK models allow high confidence in prospective DDI predictions [1]. This application of modeling and simulation has been reflected in the regular inclusion of PBPK model information into new drug application (NDA) submissions [4, 5••] and recent use in final drug product labeling text with explicit dosage recommendations (see examples below). Similarly, the simulations may support selection of dose strengths to be developed. In order to generate a well-validated PBPK model for a drug, a large amount of data need to be collected. Such data include pharmacokinetics of drug substance and metabolites, drug solubility and permeability, plasma protein binding, contributions of individual enzymes to hepatic and extrahepatic clearance, enzyme inhibition, inactivation and induction, clearance by non-metabolic routes (e.g., urinary and biliary secretion information), and any existing clinical drug-drug interaction information. Only when a good description of compound pharmacokinetics and metabolism has been established can drug-drug interaction predictions and the consequences for efficacy and safety be adequately addressed.

Improvements in in vitro technologies and the buildup of system knowledge (enzyme abundance, physiological parameters, effect of disease, age, sex, and polymorphism status) have allowed increasingly realistic computational models of the human body to be developed [11]. Confidence in competitive CYP inhibition measurement and consequent DDI prediction is typically high. In contrast, although availability, consistency, and sensitivity of time-dependent inhibition (TDI) measurement have improved considerably [12], challenges still exist in the quantitative extrapolation of TDI data. This is especially true in complex situations, for example, where time-dependent inhibition is combined with active uptake or enzyme induction. The human immunodeficiency virus (HIV) drug ritonavir, used to boost the bioavailability of antiviral agents such as saquinavir by inhibition of CYP3A4, is an example of a complex case. As well as being a CYP3A4 substrate, ritonavir inhibits, inactivates, and induces CYP3A4 [13–15]. It also inhibits and induces other drug-metabolizing enzymes [16].

The other facet of DDI assessment, that of victim DDIs, can be made with confidence for drugs principally metabolized by well-characterized metabolic enzymes (e.g., CYPs 1A2, 2C8, 2C9, 2C19, 2D6, 3A4). However, model validation is more difficult and prediction confidence is lower for enzymes such as aldehyde oxidase (AO), flavin monooxygenases (FMOs) and UGP-glucuronosyltransferases (UGTs) where human pharmacokinetic data for selective substrates and for in vivo interactions with inhibitors are lacking.

This review draws on recent Roche experiences combined with key literature examples to provide an overview of the current state of the art in DDI prediction and ongoing developments in the field. The structures of the drugs featured in this review, together with information relevant to their metabolic DDIs, can be found in Table 1.

**Table 1:**
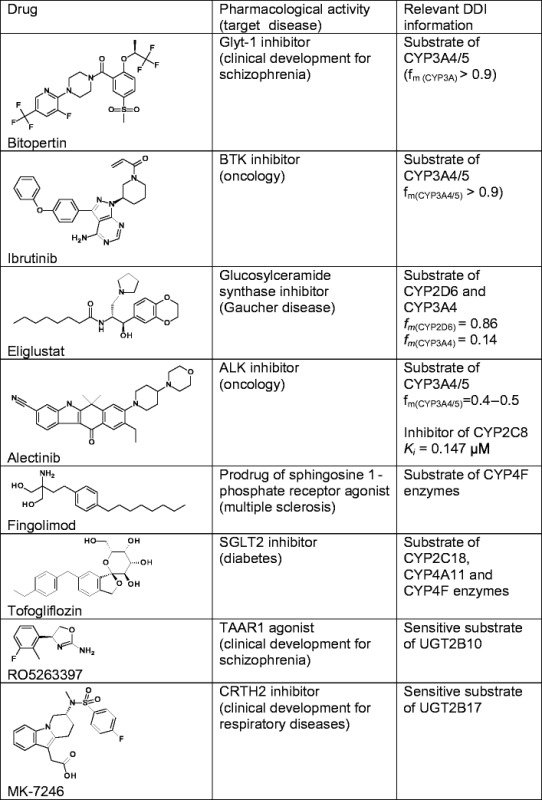
List of investigated drugs, their pharmacology and relevant metabolic DDI information

## The Recent Past: Mechanistic Understanding of DDIs Through Retrospective Modeling

### Bitopertin Case Study—Drug-Drug Interaction with CYP3A4 Inhibitors

Bitopertin inhibits the glycine transporter type 1 (GlyT1), which is expressed in the central nervous system and in peripheral tissues, mainly in erythroid cells [17, 18]. Bitopertin is cleared slowly and almost exclusively by oxidative metabolism, primarily via CYP3A4 (f_m(CYP3A enzymes)_ > 90% in vitro) with less than 0.1% of the administered dose excreted in the urine as unchanged drug [19•]. The half-life is approximately 2 days.

The pharmacokinetics of bitopertin was predicted prior to clinical studies using a PBPK model developed on the basis of non-clinical data [20]. After entry into the clinic, the model-predicted pharmacokinetics were found to be in close agreement with observations and the model was refined [21] and then applied to simulate the potential for drug-drug interactions. The clinical effect of CYP3A4 inhibition on bitopertin exposure was assessed in two studies in healthy volunteers with open-label, two-period, fixed-sequence designs [19•].

**Fig. 1:**
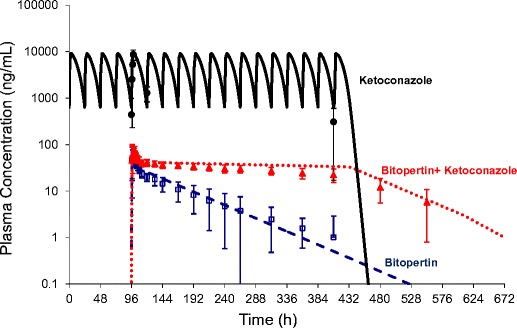
Effect of ketoconazole on exposure of bitopertin. *Symbols* are mean (±standard deviation) plasma concentration-time profiles after administration of 400 mg/day ketoconazole (*filled black circles*), bitopertin 10 mg alone (*empty blue squares*), or concurrently with ketoconazole (*filled red triangles*). The *lines* are the plasma concentrations simulated with a PBPK model in GastroPlus. Single dose of bitopertin alone (*dashed blue line*), bitopertin with ketoconazole (*dotted red line*), ketoconazole 17 days (*solid black line*)

Ketoconazole, a strong CYP3A4 inhibitor, increased the bitopertin AUC from 0 to 312 h (AUC_0–312 h_) 4.2-fold (90% confidence interval [CI] 3.5–5.0) while erythromycin, a moderate CYP3A4 inhibitor, increased the AUC from time zero to infinity (AUC_0–inf_) 2.1-fold (90% CI 1.9–2.3). The AUC_0–inf_ ratios predicted by PBPK modeling for these interactions were in good agreement at 7.7 and 1.9, respectively (note that the AUC_0–312 h_ ratio underestimated the full DDI to some extent). The effect on C_max_ was minor, <25% for both inhibitors. This was consistent with a high absolute bioavailability as simulated by PBPK for bitopertin with very limited first pass extraction in both the intestine and the liver. After discontinuation of ketoconazole, the bitopertin elimination half-life decreased, becoming similar to that observed in the absence of ketoconazole indicating the reversibility of the CYP3A4/5 inhibition (Fig. 1). For bitopertin, therefore, an excellent picture of the pharmacokinetics and a model describing the CYP3A-mediated drug-drug interactions could be developed and retrospectively validated using emerging clinical data. Details of the PBPK model can be found in Supplementary Table 1. DDI study and simulation data are also available in the Supplementary Materials.

## Current State of the Art: Interpolation and Limited Prospective DDI Prediction Gain Regulatory Acceptance

PBPK models have initially found use in incorporating the results of DDI studies into an overall description of the pharmacokinetics, then in interpolating results from DDI studies with strong probe inhibitors/inducers for the enzyme of interest (“mechanistic DDI study”) to DDIs with moderate and mild inhibitors/inducers. In addition, PBPK models have been applied to extrapolation of DDI results to subpopulations such as organ failure, geriatrics, or certain phenotypes of the involved metabolic enzymes where it is often ethically and/or practically challenging to investigate DDI [22, 23]. Such simulations have been used for guiding dose adjustment in drug labels in lieu of actual clinical study results since 2009 [1]. Ibrutinib and eliglustat are two examples selected to illustrate how a PBPK model was developed for drugs mainly metabolized by CYP3A and CYP2D6, respectively, and applied to DDI assessment which were accepted in final product labels.

### Ibrutinib Case Study—Model-Based Interpolation of CYP3A4 Inhibition DDIs

Ibrutinib is a Bruton’s tyrosine kinase inhibitor developed for treatment of leukemia. Ibrutinib is completely absorbed after oral administration and extensively metabolized in the intestine and liver mostly by CYP3A4 and lesser extent by CYP2D6 [24]. The absolute bioavailability of ibrutinib 560 mg (approved dose) was 3.9 and 8.4% in the fasted and fed states, respectively [25]. The intestinal and hepatic bioavailability (F_g_ and F_h_) evaluated after oral (140 mg) and intravenous (100 μg, ^13^C_6_ labeled) administration in the fed state were determined as 47.0 and 15.9%, respectively, in an IV microdose study with grapefruit juice pretreatment [25]. A PBPK model was developed by integrating available physicochemical properties, in vitro experiments, and clinical pharmacokinetic (PK) data [26]. The intrinsic clearance of ibrutinib in human liver microsomes was inhibited 95.8% in the presence of 1 μM of a strong CYP3A inhibitor, ketoconazole [27], and this information was incorporated into the PBPK model for DDI simulations with various CYP3A4 modulators. Capability of the PBPK model to predict CYP3A4 DDI for ibrutinib as substrate was examined by predicting fold increase in C_max_ and AUC of ibrutinib in the presence of ketoconazole and compared to the observations in the clinical study [28] (predicted vs. observed: 19- and 29- fold for C_max_ and 28- and 24-fold for AUC). Subsequently, the PBPK model was verified by showing consistency between prospectively simulated fold decrease in C_max_ and AUC of ibrutinib in the presence of a CYP3A inducer, rifampicin, and the observations [28] (predicted vs. observed: 11- and 13-fold for C_max_ and 10- and 10-fold for AUC). The verified PBPK model was then used to simulate unstudied clinical DDIs with mild (fluvoxamine, azithromycin), moderate (diltiazem and erythromycin), and strong (voriconazole, clarithromycin, itraconazole) CYP3A inhibitors to guide dose reduction from 560 to 140 mg in concurrent administrations with moderate CYP3A4 inhibitors.

The PBPK model simulations of DDI with moderate (efavirenz) and strong (carbamazepine) CYP3A inducers supported ibrutinib dose of 560 mg in co-administrations with moderate CYP3A inducers since predicted exposure was within defined therapeutic exposure range [27]. The DDI risk assessment and dose modification guidance for ibrutinib based on the PBPK modeling and simulations were submitted in new drug applications and approved in the USA [27, 29], Canada [30], European Union [31], and Japan [32] and used in drug labels.

### Eliglustat Case Study—Model-Based Extrapolation to Polymorphic CYP2D6 Phenotype Individuals

Eliglustat is an oral glucosylceramide synthase inhibitor and indicated to treat symptoms of Gaucher disease type 1 [33]. This drug is extensively metabolized by CYP2D6 (f_mCYP2D6_ = 86%) and to a lesser extent by CYP3A4 (f_mCYP3A4_ = 14%). Clinical DDI investigations in CYP2D6 intermediate metabolizers (IMs) and extensive metabolizers (EMs) showed increase in AUC of eliglustat by approximately 5-fold (IMs) to 10-fold (EMs) when co-administered with paroxetine (strong CYP2D6 and weak CYP3A4 inhibitor) and by 4-fold (in both IMs and EMs) when co-administered with ketoconazole. Eliglustat is a time-dependent inhibitor of CYP2D6 and multiple dose PK exhibited dose- and time-dependent behavior. Multiple doses of eliglustat increased AUC of metoprolol (CYP2D6 substrate) by approximately 2-fold in EMs and IMs. A PBPK model of eliglustat was developed and its ability to describe PK in different CYP2D6 phenotypes including poor metabolizers (PMs) and to predict clinical DDIs was confirmed. The PBPK model was then used for predicting DDIs with moderate inhibitors of CYP2D6 (terbinafine) and CYP3A4 inhibitors (fluconazole) in EMs and IMs. Moreover, DDIs with moderate to strong CYP3A4 inhibitors (fluconazole and ketoconazole) in PMs were predicted using the PBPK model since CYP3A4 inhibition effect on eliglustat has not been clinically investigated in PMs. Predicted fold increase in AUC_0–24 h_ of eliglustat in concomitant administration with ketoconazole in PMs was 6.2 [34], higher than that in EMs and IMs, due to higher dependency on elimination through CYP3A4 metabolism, and concomitant use with strong CYP3A inhibitors is contraindicated in this population. The PBPK model enabled not only interpolations from DDI with strong enzyme inhibitors to the moderate inhibitors but also extrapolations of DDIs to other CYP2D6 phenotypes which complemented DDI risk assessments of eliglustat as a dual CYP2D6 and CYP3A4 substrate across CYP2D6 phenotypes. Dosage adjustment guidance in the drug label approved by FDA [34] based on these clinical studies and PBPK model predictions are summarized in Supplementary Table 2.

### Repaglinide Case Study—Model-Based Prediction of Insignificant DDI Effect to Support Appropriate Dosing Recommendations

As a clinically relevant probe substrate, repaglinide is commonly used to assess the DDI risk for CYP2C8 inhibitors. Repaglinide is an antidiabetic drug whose metabolism is mediated by CYP2C8, CYP3A4, and to a lesser extent UGT enzymes [35, 36]. For assessing the DDI risk, therefore, assigning the appropriate f_m(CYP2C8)_ value for repaglinide is of great importance given the sensitivity of predicted AUC ratios to f_m_ values [37]. CYP2C8 and CYP3A4 have been reported to contribute equally to the in vitro metabolism of repaglinide, ∼50% [36]. However, an alternative f_m(CYP2C8)_ value of 0.83 has been proposed based on meta-analysis of in vivo data [38]. As these two f_m>_ values would result in very different maximal repaglinide DDI effects assuming complete enzyme inhibition (2.4 and 5.9 for f_m_ values of 0.59 and 0.83, respectively, following oral administration), it was important to consider both possibilities in the DDI assessment.

A number of clinically relevant DDIs with repaglinide have been reported (Table 2). These DDIs include interactions with inhibitors of CYP2C8, CYP3A4, and OATP1B1/3 as well as compounds which interact via multiple mechanisms. The extent of clinical DDIs with repaglinide may be assessed as (1) large extent (≥5-fold AUC change) due to inhibition of multiple processes or TDI of CYP2C8 [39, 40] and (2) a substantially lower risk can be anticipated for inhibition of a single process, <2.5-fold AUC change for competitive CYP3A4 or CYP2C8 inhibitors [41, 42].

**Table 2:** Clinical drug-drug interactions with repaglinide as victim drug available in the University of Washington DDI database

Perpetrator	AUC change (%)	Dose (mg)	*K*_i_ (μmol/L)			Mechanism	Refs
			CYP2C8^a^	CYP3A4^a^	OATP1B1/3^b^		
Gemfibrozil + Itraconazole	1830	600 + 100	Detailed below	Detailed below	Detailed below	CYP2C8, OATP1B1, and CYP3A4	[39]
Gemfibrozil	443–726	300–900	36 (9.3–87)	171 (184–406)	36 (13–68)	CYP2C8 (TDI) and OATP1B1	[38, 93, 39, 94–96, 40]
Gemfibrozil-glucuronide			*K*_*I*_ = 29, *k*_inact_ = 0.071/min	n.r.	9.3–23		
Clopidogrel	295–408	75–300	2.8–50	*K*_*I*_ = 26, *k*_inact_ = 0.053/min	4.0	CYP2C8 (TDI), OATP1B1	[97]
Clopidogrel-acyl-glucuronide			*K*_*I*_ = 9.9, *k*_inact_ = 0.047	n.r.	11–34		
Cyclosporine	143	100	n.r.	3.2 (0.3–37)	0.019–0.032 (after pre-incubation)	OATP1B1, (CYP3A4)	[98]
Teriflunomide	142	14–70	0.1	n.r.	7.1	CYP2C8, (OATP)	[99]
Telithromycin	77	800	15	87	11–121	CYP3A4	[42]
Trimethoprim	63	160	8.5	n.r.	n.r.	CYP2C8	[41]
Clarithromycin	42	250	n.r.	*K*_*I*_ = 13.1 (0.85–37.4), *k*_inact_ = 0.058 (0.0192–0.14)	8.26	CYP3A4 (TDI), OATP	[100]
Itraconazole	41	100	31	0.042 (0.0013–3.12)	n.r.	CYP3A4	[39]
Grapefruit juice	21	n/a	n.r.	TDI	n.r.	Intestinal metabolism	[101]

Data in parenthesis represent the reported range

All data are available from https://www.druginteractioninfo.org [102]

*n.r*. not relevant, *TDI* time-dependent inhibition, *n/a* not applicable

^a^Microsomal data

^b^Data from HEK, or MDCK-transfected cell lines or human hepatocytes

Alectinib (Alecensa®) is a small molecule kinase inhibitor which has received FDA accelerated approval for the treatment of patients with anaplastic lymphoma kinase (ALK)-positive metastatic non-small cell lung cancer (NSCLC) who have progressed on or are intolerant to crizotinib treatment [43]. Alectinib has shown weak competitive and time-dependent inhibition of CYP3A4 in vitro which has not translated in vivo [44]. Alectinib is also a competitive inhibitor of CYP2C8 with an unbound in vitro *K*_i_ value of 0.147 μM [45]. DDI predictions with repaglinide were performed using a PBPK modeling approach to evaluate the clinical relevance of the CYP2C8 liability. The measured f_u(plasma)_ and blood to plasma concentration ratio used in the PBPK simulations were 0.003 and 2.64 (consequently the f_u(blood)_ was 0.0011). In the PBPK assessment of repaglinide, DDI potential f_m(CYP2C8)_ values of both 0.59 and 0.83 were used. In order to investigate the sensitivity of the DDI simulations to the in vitro *K*_i_ value of alectinib, the following scenarios were tested for both repaglinide models: true in vivo *K*_i_ = 1×, 1/3×, 1/10×, and 1/30× of the in vitro *K*_i_ value [46].

Based on the simulations, no significant interaction (>25% change of AUC) is anticipated regardless of the assumptions around the in vivo f_m(CYP2C8)_ value of repaglinide (0.59 or 0.83). A sensitivity analysis revealed that a risk for an AUC change of greater than 25% can only be expected in case that the in vivo inhibitory potency of alectinib is considerably higher than anticipated from in vitro data and the in vivo f_m(CYP2C8)_ of repaglinide is 0.83. This model-based assessment for characterization of clinical DDI between alectinib and CYP2C8 substrates was accepted in lieu of a clinical DDI study with repaglinide and justified the product labeling text “No clinical meaningful effect on the exposure of … repaglinide (sensitive CYP2C8 substrate) is expected following co-administration with ALESENSA” [43].

### Alectinib Efficacy Case Study—Translation of DDI Effects Into Pharmacodynamic Effects: Relevance and Contribution of a Major Active Metabolite to Analysis and Interpretation of a Clinical DDI

Human metabolites are usually considered in terms of safety when formed at greater than 10% of total drug-related systemic exposure at steady state [47]. In terms of drug-drug interactions, metabolites formed in vivo and reaching significant exposures (e.g., ≥25% of parent drug exposure) have been recommended to be characterized further in terms of metabolism, transport, and for potential drug-drug interactions [48]. A metabolite may bind to on- or off-target receptors and thus can be considered active and contribute to intended and/or unintended effects [49–51].

Alectinib is metabolized by CYP3A4 and to a smaller extent by other enzymes to generate a number of metabolites including a major metabolite M4 [52]. Population PK analysis of the pivotal phase 2 studies showed that the geometric mean M4 metabolite/parent (M/P) ratio in plasma was 0.4 with an effective elimination half-life (*t*_1/2_) of approximately 33 and 31 h for alectinib and M4, respectively [53]. In vitro pharmacology studies demonstrated that both alectinib and M4 are potent inhibitors of the target ALK with similar potency (IC_50_ of 1.9 and 1.2 nM, for alectinib and M4, respectively, in biochemical assays) and exhibit similar plasma protein binding (>99% protein bound) [52].

**Fig. 2:**
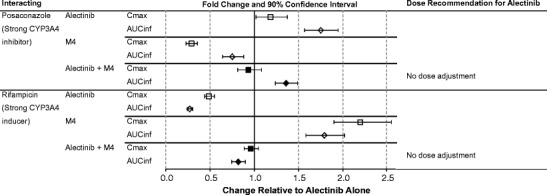
Forrest plot of the drug-drug interaction potential between alectinib and the potent CYP3A inhibitor, posaconazole, or the potent CYP3A inducer, rifampin [48]

As both alectinib and M4 are substrates of CYP3A, dedicated clinical pharmacology studies were undertaken to evaluate the effect of a strong CYP3A inhibitor (posaconazole) and strong CYP3A inducer (rifampin) on the pharmacokinetics of alectinib and M4 [44]. Notably, the results from the clinical DDI study with posaconazole showed that its co-administration increased alectinib exposure and decreased M4 exposure while results from the rifampin DDI study showed that its co-administration decreased alectinib exposure and increased M4 exposure [44] (Fig. 2). As both alectinib and M4 are similarly active against ALK and exhibit similar protein binding, it is expected that both substances contribute to overall alectinib efficacy and safety. Therefore, to support clinical dosing recommendations in the presence of CYP3A inhibitors and inducers, changes in the combined molar exposure of alectinib and M4 (i.e., molar sum of alectinib + M4) were evaluated (Fig. 2). The minor effects seen on the combined exposure supported the statement “no dosage adjustment required with co-administered CYP3A inhibitors or inducers” in US prescribing information for Alecensa® [43].

The alectinib case represents an approach to the understanding of drug-drug interaction potential by utilization of integrated non-clinical and clinical data of a parent molecule and its major active metabolite. The knowledge of clinical pharmacology attributes of both the parent and metabolite enabled dosing recommendations based on the changes occurring to both substances. To support this, characterization of both alectinib and M4 was undertaken throughout the development process from preclinical safety and drug metabolism/pharmacokinetic testing through to clinical exposure-response evaluation of alectinib [54, 55]. Indeed, clinical exposure-response analyses evaluated the relationship between key efficacy and safety endpoints emerging from alectinib pivotal studies and the combined exposure of alectinib and M4 [53]. Thus, while the changes seen in the alectinib exposure when co-administered with posaconazole or rifampin may have potentially warranted dosage adjustments, consideration of the combined changes suggested that no dosage adjustments were needed. This approach to consideration of parent and metabolite contributions to clinical DDI or exposure-response interpretation has been successfully applied previously for other small molecules with active metabolites (e.g., regorafenib, ezetimibe, ruxolitinib, dabrafenib, and sunitinib) [56–61]. Cumulatively, the alectinib case illustrates the relevance and contribution of a major active metabolite to clinical DDI analyses and interpretation.

## Current Frontiers in DDI Prediction From In Vitro

### Enhanced DDI Predictions From Time-Dependent Inhibition Measurements Using Human Hepatocytes Suspended in Full Plasma

Preclinical prediction of CYP inhibition-mediated DDIs has been performed conventionally using the well-characterized and intensively studied human liver microsomal (HLM) assay, which shows high detection sensitivity and low likelihood of false-negative predictions [62]. An in vitro assay using human hepatocytes (hHEPs) suspended in whole human plasma (plasma hHEPs) has been reported to give more accurate prediction of the extent of clinical relevant effect due to CYP inhibition [63–66]. Advantages of assessing DDI in human hepatocytes supplemented with 100% plasma include (1) inherent accounting for plasma protein and microsomal/hepatocyte binding of a drug, (2) compound is available to enzyme in its native environment within the cell, i.e., more a physiologically relevant condition, (3) metabolism of the compound by both CYP and non-CYP pathways is possible, and (4) transporter-mediated uptake into hepatocytes may occur.

An elegant study recently published by Mao et al. [67••] compared side-by-side DDI prediction due to CYP3A inhibition from the plasma hHEP assay with three other assays: (a) HLM, (b) plated hHEPs, and (c) hHEPs suspended in Dulbecco’s modified Eagle’s medium (DMEM) for 12 marketed drugs (10 protein kinase inhibitors and 2 prototypical CYP3A time-dependent inhibitors). Kinetic parameters were generated for the apparent reversible inhibition constant (*K*_*i,*app_) and/or TDI (*K*_*I,*app_ and *k*_inact_) and directly used for quantitative prediction of the fold-increase in midazolam AUC_0–inf_ (AUC_R_) following co-administration with CYP3A inhibitors based on a static mechanistic model and the total average systemic plasma concentration without correction for free drug fraction (f_u_). The result from this study demonstrated that the plasma hHEP assay offered a clear enhancement of DDI prediction (95% accuracy) with no false-negative or false-positive outcomes. The accuracies for the other three assays were 58, 84, and 74% for HLM, plated hHEPs, and DMEM hHEPs, respectively.

In this study [67••], a number of drugs were shown to give both reversible inhibition and TDI for CYP3A in the HLM assay (for example erlotinib, nilotinib, and pazopanib) but interestingly, these drugs were not inhibitory in the plasma HEP assay. While the clinical data confirmed low DDI due to CYP3A inhibition for these drugs as predicted by the plasma HEP assay, a more complete mechanistic understanding for the discrepancy between the two systems would be helpful when considering the differential sensitivities of the test systems. The traditional HLM TDI assay is robust, sensitive, and backed by a substantial body of published data [12, 68, 69] which can be used to rank and to some extent to predict CYP-mediated DDI during the discovery stage. It is however suggested to consider using the plasma hHEP TDI assay for an enhanced assessment of the potential DDI during the candidate selection and early stages of drug development as a de-risking approach for TDI-positive candidate compounds.

### Challenges of DDI Prediction in Cases of Metabolism by “Unusual” CYP Enzymes or Non-CYP Enzymes

Despite advances in in vitro enzymology technologies, there continues to be much to learn about enzymes which, while unimportant in the metabolism of drugs in general, are key contributors to the metabolism of particular drug compounds. For example, the SGLT2 inhibitor tofogliflozin is metabolized by CYPs 2C18, 4A11, and 4F3B [70], and the multiple sclerosis drug fingolimod is metabolized by CYP4F enzymes [71]. These enzymes are usually regarded as “minor” CYP isoforms and would not routinely be included in enzyme phenotyping screens [10•, 72]. This raises the question of how one is to know that an important pathway is “missed” in initial in vitro assessments. Due to the availability of well-characterized and selective inhibitors for CYP isoforms, it may be apparent should the activities of routinely tested CYP enzymes not account for the majority of metabolism in vitro. In such cases, additional in vitro work using recombinantly expressed enzymes and (semi-)selective CYP inhibitors may be performed to try to obtain more clarity on enzyme contributions to metabolism, although this may prove challenging. DDI risks could then be addressed through screening of potential co-medicant substances either as inhibitors of the involved metabolic enzyme or, more empirically, as inhibitors of turnover of the drug in development itself. Due to a lack of system information (enzyme expression and activity levels, polymorphism status, effect of disease, ontogeny), it is unlikely that special population or polymorphism risk assessments can be made at this time.

The situation is even more challenging when “unusual” *non-CYP* enzymes are involved. In one recent example, an investigational trace amine-associated receptor antagonist RO5263397 was found to be principally cleared by UGT2B10 [73••]. At the time of compound selection, UGT2B10 was not considered an important enzyme in drug metabolism and was not commercially available for testing, and no selective inhibitors were characterized [74–77]. Co-administration with potent UGT2B10 inhibitors could potentially mimic the UGT2B10 poor metabolizer phenotype which resulted in a 136-fold higher AUC for one individual after a single 10 mg dose in a phase I clinical study [73••].

Such cases also provide substantial learning opportunities. As a result of this observation, a new splice site polymorphism was identified (prevalent in individuals of African origin but almost absent in Caucasians). This is relevant for clearance of other UGT2B10 substrates [78, 79]. In addition, increased understanding of the enzyme system and in vitro tools to assess UGT2B10 contribution to metabolism have been developed which can be rapidly employed in the future. In this way, UGT2B10 illustrates the process by which an enzyme not previously considered in drug metabolism testing progresses from being an “essentially uncharacterized” to a “largely characterized” metabolic enzyme system [80, 81]. A similar experience had been reported by Wang et al. for a Merck development compound MK-7246 which is cleared by polymorphic UGT2B17 [82]. It is likely that such learning experiences will be repeated as drug development continues to move into areas of novel chemical space in pursuit of new drug targets and further examples are discovered where previously little studied enzymes are important for individual drug clearance.

## Future Prospects for DDI Prediction

To date, most in vitro systems used in DDI prediction have employed short timescale incubations to generate mechanistic parameters which can then be used to build up long-term model predictions of DDIs in vivo. Short timescale incubations cannot however address issues such as enzyme inactivation by highly metabolically stable compounds or the interplay of enzyme inactivation and induction which will drive the effective steady-state change in metabolic enzyme capacity. Although the sensitivity of short-term plated human hepatocytes to inhibition and induction has been demonstrated [83], such systems are unlikely to reflect steady-state conditions due to the transient nature of the cell cultures used. The advent of long-term hepatocyte culture systems may allow effective in vitro pharmacokinetic assessments to be made which will better reflect the clinical situation. The potential of long-term hepatocyte cultures has initially been demonstrated for clearance assessment of metabolically stable compounds [84–87••]. Their application to more sophisticated ADME assessments, such as induction [88], the effect of active uptake on apparent induction potency [89•], metabolism profiling/cross-species comparison [90], and to a limited extent for drug-drug interactions [85] have also been demonstrated. New long-term hepatocyte systems may therefore offer a completely new opportunity to simultaneously study multiple processes involved in drug-drug interactions which were not previously possible in vitro, especially for highly metabolically stable compounds. The development of long-term hepatocyte systems may also be seen as a first step in the direction of functional in vitro test systems with cells from multiple organs such as the liver, intestine, kidney, skin, and brain [91, 92], within a single test system (“chip”). When validated, data from the new experimental systems will quickly be incorporated into PBPK-based modeling tools further enhancing prediction of clinical DDIs.

## Conclusions

This review has drawn upon personal experiences and recent literature reports to highlight achievements and ongoing challenges in the rapidly developing areas of metabolic DDI assessment, prediction, interpretation, and drug product labeling. Examples have been shown of how a model-based approach to understanding DDIs has progressed from data integration (bitopertin) to being accepted for interpolative (ibrutinib) and increasingly extrapolative DDI predictions (eliglustat and alectinib). Scientific confidence in and regulatory acceptance of PBPK modeling have increased with growing knowledge of DDIs, availability and robustness of in vitro test systems, and experience in DDI prediction. Predictions from well-executed analyses using validated models have enabled explicit dosing recommendations in product labels for clinical DDIs based on PBPK modeling in lieu of dedicated clinical DDI studies. Modeling approaches may indeed offer the only way to explore some potential DDIs where clinical investigation is unfeasible due to ethical considerations or the inability to recruit suitable study subjects.

The impact of characterizing major active metabolites during drug development has also been exemplified in the case of alectinib. This has been shown to be critical in the interpretation of clinical DDIs where exposure changes occur to both the parent and an active metabolite and are relevant to clinical efficacy and safety. Understanding the pharmacological, pharmacokinetic, and disposition properties of a metabolite using in vitro and in vivo studies can allow for estimation of its contribution in clinical DDI interpretation and subsequently its potential impact on clinical efficacy and safety in support of appropriate dosing recommendations.

A sometimes underemphasized factor affecting DDI predictions is the availability of good quality clinical DDI data with which can be used for validation purposes. This is especially the case for drugs predominantly metabolized by “unusual” CYP enzymes or non-CYP enzymes. Examples where CYP4F enzymes or UGT2B10 catalyze drug clearance have been discussed. We can expect that the continued development of experimental techniques and the increases in knowledge of enzymology and DDI will be reflected in increased DDI prediction confidence for such drugs.

Use of a whole plasma human hepatocyte TDI assay has shown to improve in vitro-in vivo extrapolation. Further TDI assay developments are anticipated using long-term hepatocyte culture systems and other “organs on a chip” technologies. These offer the promise of in vitro systems where an integrated assessment of enzyme inhibition, inactivation, and induction can be made. The ability to use the same modeling approaches to understand such “in vitro pharmacokinetics/DDI” experiments and then directly transfer this understanding to the human DDI situation may allow a further step forward in DDI prediction to be made in the near future.

## Electronic Supplementary Material

ESM 1 (DOC 63 kb)

ESM 2 (DOCX 16 kb)

ESM 3 (XLSX 113 kb)
