# Supplementary material for: Progress in Prediction and Interpretation of Clinically Relevant Metabolic Drug-Drug Interactions: a Minireview Illustrating Recent Developments and Current Opportunities
Source: Curr Pharmacol Rep. 2017 Feb 1;3(1):36–49. doi: 10.1007/s40495-017-0082-5 (PMC5315728; doi:10.1007/s40495-017-0082-5)
Supplement: Supplementary file 1 — (DOC 63 kb) [file 40495_2017_82_MOESM1_ESM.doc]

**Supplementary Table 1: Additional Data for PBPK models**

**Software versions**

The PBPK disposition model equations for human have been previously described in *Poulin, P. and F. Theil (2002). "Prediction of pharmacokinetics prior to in vivo studies II. Generic physiologically based pharmacokinetic models of drug disposition." J. Pharm. Sci. 91: 1358-1370.*

All simulations included in the paper were made with models which have been transferred into GastroPlus version 8.0 to provide integrated PBPK absorption and disposition models.

**Oral absorption simulations**

The model for Bitopertin has been described in *Parrott et al. (2014). "Physiologically Based Absorption Modelling to Predict the Impact of Drug Properties on Pharmacokinetics of Bitopertin." The AAPS Journal 16(5): 1077-1084*. The basic absorption inputs for the model are tabulated below.

|  | Refined Model |
| --- | --- |
| Dosage form: | IR: Capsule |
| Dose Volume = | 250 mL |
| Reference LogD = | 3.03 |
| pH Reference for LogD = | 7.4 |
| Diffusion Coefficient = | 0.559cm^2^/s*10E5 |
| Drug particle density = | 1.2 g/mL |
| Reference Solubility = | 0.025 mg/mL |
| Reference Solubility pH = | 6.6 |
| Dissolution Model: | Johnson |
| Adjust solubility/dissolution for NanoParticle effect: | OFF |
| Adjust solubility for Bile salt effect: | OFF |
| Adjust diffusion coefficient for Bile salt effect: | OFF |
| Mean Drug Particle Radius = | 6 microns |
| Precipitation options: Form new particles with radius: | 1 |
| Precipitation Time = | 900 s |
| Primary Permeability = | 3.5 cm/s2 |

**Key input data for PBPK modelling of bitopertin drug interaction.**

To allow for the modelling of drug–drug interactions it was necessary to assign the clearance of bitopertin to specific enzymes and this was done assuming that CYP3A4 is the only enzyme responsible for metabolism in the liver and gut. The model parameters used for bitopertin intestinal metabolism and for the inhibition of CYP3A4 enzyme by ketoconazole are given below.

|  | **Bitopertin** | **Ketoconazole** |
| --- | --- | --- |
| Fraction escaping gut, first-pass | 0.97 |  |
| Competitive: K_i_, unbound, μM ^a^ |  | 0.0015 |

^a^ Kato M, Shitara Y, Sato H, Yoshisue K, Hirano M, Ikeda T, et al. The quantitative prediction of CYP-mediated drug interaction by physiologically based pharmacokinetic modeling. Pharm Res. 2008;25(8):1891–1901.

.
