# Supplementary material for: Progress in Prediction and Interpretation of Clinically Relevant Metabolic Drug-Drug Interactions: a Minireview Illustrating Recent Developments and Current Opportunities
Source: Curr Pharmacol Rep. 2017 Feb 1;3(1):36–49. doi: 10.1007/s40495-017-0082-5 (PMC5315728; doi:10.1007/s40495-017-0082-5)
Supplement: Supplementary file 2 — (DOCX 16 kb) [file 40495_2017_82_MOESM2_ESM.docx]

**Supplementary Table 1** Summary of recommended dosage adjustment of eliglustat in the drug label (FDA 2014a)

| **Concomitant medications** | **CYP2D6 phenotype^a^** | | |
| --- | --- | --- | --- |
|  | **EM** | **IM** | **PM** |
| Without concomitant medications | 84 mg b.i.d. | 84 mg b.i.d. | 84 mg q.d. |
| Strong or moderate CYP2D6 inhibitors concomitantly with strong or moderate CYP3A inhibitors | Contraindicated | Contraindicated | — |
| Strong CYP2D6 inhibitors | 84 mg q.d. | 84 mg q.d. | — |
| Moderate CYP2D6 inhibitors | 84 mg q.d. | 84 mg q.d. | — |
| Strong CYP3A inhibitors | 84 mg q.d. | Contraindicated | Contraindicated |
| Moderate CYP3A inhibitors | 84 mg q.d. | Not recommended | Not recommended |
| Weak CYP3A inhibitors | — | — | Not recommended |

^a^ Guidance indicated in gray box is based on PBPK model simulations.

EM: extensive metabolizers; IM: intermediate metabolizers; PM: poor metabolizers.
